# An analysis of prognostic factors in a cohort of low-grade gliomas and degree of consistency between RTOG and EORTC scores

**DOI:** 10.1038/s41598-022-20429-8

**Published:** 2022-09-30

**Authors:** Isaura Fernández Pérez, Diana Valverde, Concepción Fiaño Valverde, Jenifer Brea Iglesias, María José Villanueva Silva, Martín Lázaro Quintela, Bárbara Meléndez

**Affiliations:** 1 Department of Medical Oncology, University Hospital Complex of Vigo, Vigo, Spain; 2 Rare Disease Research Group, Galicia Sur Health Research Institute (IISGS), Faculty of Biology, University of Vigo, Vigo, Spain; 3 Department of Pathology, University Hospital Complex of Vigo, Vigo, Spain; 4 Translational Oncology Research Group, Galicia Sur Health Research Institute (IISGS), Vigo, Spain; 5 Molecular Pathology Research Unit, Department of Pathology, University Hospital of Toledo, Toledo, Spain

**Keywords:** Cancer, Neuroscience, Diseases, Medical research, Oncology

## Abstract

Due to their rarity and heterogeneity and despite the introduction of molecular features in the current WHO classification, clinical criteria such as those from the European Organization for Research and Treatment of Cancer (EORTC) and the Radiation Therapy Oncology Group (RTOG) are still being used to make treatment decisions in low-grade gliomas (LGG). Patients with diffuse low-grade glioma treated at our institution between 2002 and 2018 were analyzed, retrieving and assessing the degree of consistency between the EORTC and RTOG criteria, as well as the *isocitrate dehydrogenase 1 and 2* (IDH) gene mutational status. Likewise, multivariate analyses were performed to ascertain the superiority of any of the factors over the others. One hundred and two patients were included. The degree of consistency between the RTOG and EORTC criteria was 71.6% (K = 0.426; p = 0.0001). Notably, 51.7% of those assigned to low risk by the EORTC were classified as high risk according to the RTOG classification. In multivariate analysis, only complete resection, age > 40 years, size and IDH mutation status were independently correlated with OS. When the RTOG and EORTC scores were entered into the model, only the EORTC model was independently associated with mortality. The degree of consistency between the EORT and RTOG criteria is low. Therefore, there is a need to integrate clinical-molecular scores to improve treatment decisions in LGG.

## Introduction

Low-grade infiltrating gliomas (LGGs) are a rare entity, accounting for 15–20% of brain gliomas, and are a heterogeneous group of tumors characterized by a slow growth rate and a low mitotic index^1^. LGGs arise mainly in adults between 20 and 40 years of age, with neurological symptom onset, the most common being seizures (80%), headache and motor deficit symptoms^2^.

Both the European Organization for Research and Treatment of Cancer (EORTC) and the Radiation Therapy Oncology Group (RTOG) developed two different prognostic scores based on clinical risk factors to classify LGG and help clinicians make therapeutic decisions (Table 1).

**Table 1:** EORTC and RTOG scores.

EORTC adverse prognostic factors^5^	RTOG adverse prognostic factors^4^
Age ≥ 40 Tumor diameter ≥ 6 cm Tumor crossing midline Astrocytoma histology Pre-operative neurological deficit	Age ≥ 40 Incomplete resection
High risk group: presence of three or more risk factors	High risk group: presence of one or more risk factors

According to the RTOG criteria, patients are classified as low risk provided both that they are younger than 40 years of age and that their tumor has been completely resected. Otherwise, (age ≥ 40 or incomplete resection), they are deemed to be high-risk patients^4^.

The criteria developed by the EORTC group are based on five items: age ≥ 40 years, presence of neurological deficits prior to surgery, tumor diameter ≥ 6 cm, tumor crossing the midline and astrocytoma histology. Patients with up to two criteria are classified as low risk; otherwise, they are assigned to the high-risk group^5^.

Until recently, histology and clinical classification were the only means to characterize these tumors, although some grade II gliomas were known to behave as grade IV gliomas, and conversely, certain grade IV gliomas had a more indolent behavior. In 2016, the World Health Organization (WHO) incorporated molecular parameters into the classification of LGG for the first time^3^. The IDH (*isocitrate dehydrogenase 1 and 2)* gene mutational status and codeletion of the 1p and 19q whole arms are critical markers defining entities.

Regarding treatment, following surgery, the addition of radiotherapy and chemotherapy has been proven to increase disease-free survival (DFS) and overall survival (OS) in clinical trials^4,5^, thus becoming the current standard treatment. Nonetheless, one should acknowledge the fact that the results of those trials may not be fully applicable to the LGG molecular-defined population, which might not be superimposable to the ones in which the RTOG^4^ or EORTC^5^ inclusion criteria were used. Moreover, owing to the slow growth rate and the young age of onset, careful attention should be paid to treatment side effects, notably radiotherapy-induced late cognitive impairments as well as infertility and second neoplasms caused by chemotherapy. Therefore, despite the improvement of knowledge of the molecular landscape of LGG, treatment strategies are still a matter of debate^6,7^.

In the fifth edition of the WHO classification, the diagnosis of adult IDH wild-type (IDHwt) diffuse gliomas is strongly based on molecular parameters, and even those that histologically do not present glioblastoma features but present one of three genetic parameters, *TERT* promoter mutation, *EGFR* gene amplification, or combined gain of entire chromosome 7 and loss of entire chromosome 10 [+ 7/ − 10], will also be classified as glioblastoma^8^.

In addition, in the new classification, diffuse astrocytic tumors with IDH mutation are considered a single type and are classified as malignancy grade 2, 3 or 4. In these tumors, the presence of the homozygous deletion of CDKN2A/B, which has a worse prognosis, determines a WHO malignancy grade of 4, even in the absence of all required morphological parameters, e.g., in the absence of microvascular proliferation or necrosis. This separation of astrocytomas into IDHwt and mutated type tumors implies that all hospitals should have access to adequate molecular tests to identify cases of astrocytomas with noncanonical IDH mutations that will not be detected by IDHR132H.

Immunohistochemistry. It further implies that the complementary treatments we are currently applying based on clinical criteria are outdated.

Molecular classification helps us to differentiate GBG from high-grade GBG and to classify patients into prognostic groups, but prospective and randomized studies would be needed to indicate a change in treatment based on molecular findings, and currently in most centers, the classic clinical criteria continue to be applied, in particular the RTOG criteria, which are those used for the indication of complementary treatments in GBG according to the RTOG 9802 study^4^.

To assess the prognostic value of clinical scores as well as the degree of consistency with molecular analysis, a retrospective study of patients diagnosed and treated between 2002 and 2018 at our academic institution was carried out, with a double objective: to analyze the degree of consistency between EORTC and RTOG criteria in our environment and to establish whether the addition of molecular criteria of the current WHO classification provides any benefit as a prognostic tool.

This publication presents the results of the assessment of the degree of concordance between the EORTC and RTOG criteria, as well as the prognostic impact of each risk factor as an independent variable.

## Materials and methods

### Patients

This study included all patients diagnosed with LGG according to the WHO 2016 classification and treated between 2003 and 2018 at our institution. Data were reviewed from medical records (electronically based since 2009). Notably, patients with LGG as defined as histologic grade 2 glioma, irrespective of IDH mutation or 1p/19q status, were included. Only patients with enough data to assess their risk according to both EORTC and RTOG criteria were included in the analysis.

Histological diagnosis was reevaluated, and tumors were reclassified in accordance with the 2016 WHO classification and suggested changes in the most recent WHO classification 2021^8^.

Surgical treatment type and the extent of surgery were defined according to surgical report and by the findings of the first MRI performed after surgery. Patients were divided into three groups according to the type of surgery, namely, biopsy only, partial surgery or complete surgery.

Data were collected on the treatment strategy received after surgery, grouping them into three categories: radiotherapy only, radiotherapy plus chemotherapy or no adjuvant treatment at all.

Overall survival was calculated from the date of biopsy to the date of death or the last follow-up date, whichever occurred first.

Data regarding age, sex, performance status and neurological deficits at diagnosis, extent of resection, histology type and molecular data of patients in whom 1p19q codeletion or IDH mutational status was performed were collected from electronic or paper medical records.

We calculated the risk of each patient according to the analysis of the patient variables required by each score (EORTC, RTOG).

The study was approved by the Galician Bioethics Committee on December 12, 2019, and all research was performed in accordance with the Declaration of Helsinki. Informed consent was obtained from all surviving patients.

### Molecular analyses

Molecular analyses were performed on formalin-fixed paraffin-embedded (FFPE) tissues. Immunohistochemical staining was used to detect the IDH1 R132H mutation by using a mouse monoclonal antibody (DIA H09, dilution 1:100; Dianova GmbH, Hamburg, Germany) following the manufacturer’s recommendations. The 1p19q codeletion status was performed in LGGs deemed to be oligodendrogliomas. Fluorescence in situ hybridization (FISH) was performed on tumor-selected areas to detect chromosomal codeletion of 1p and 19q using the probes Vysis LSI 1p36 SpectrumOrange/1q25 SpectrumGreen Probes and LSI 19q13 SpectrumOrange/19p13 SpectrumGreen Probes (Abbott Laboratories, Madrid, Spain).

### Statistical analysis

Overall survival (OS) with a 95% confidence interval (95% CI) was calculated using the Kaplan–Meier method. Univariate mortality analyses were performed with the log-rank test method. Two-tailed p-values less than 0.05 were considered significant.

The Cox regression model was used for multivariate survival analyses. The log-rank test was used in all subgroups.

Cohen's kappa was used to measure the degree of consistency among the high-risk classifications derived from the EORTC and RTOG scores.

### Ethical aspects

Galicia's ethics committee approved the protocol for collecting patient data. All living patients signed the corresponding informed consent form. The authors have followed the principles outlined in the Helsinki Declaration for human or animal research. Informed consent was obtained from the live patients.

## Results

Between January 2003 and December 2018, 102 patients with low-grade gliomas were retrieved from pathological and medical records, of whom 60 were male, with a median age of 45 years (range 18–80) (Table 2). The median follow-up of the patients was 192 months, and the median OS was 162 months.

**Table 2:** Patient demographics.

Characteristics	N	Rate
Number of patients	102	
Median age (range)	45 (18–80)	
**Male**	60	58.8%
**Surgery**		
Biopsy	32	31.4%
Partial resection	34	33.3%
Complete resection	36	35.3%
**Histology**		
Astrocytoma IDH 1 mutant^a^	39	38.2%
Astrocytoma IDH1 wild type	14	13.7%
IDH1 unknown (NOS)	11	10.7%
Oligodendroglioma IDH 1 mutant + 1p19q codeleted	28	27.4%
Oligodendroglioma NOS (IDH1 unknown or 1p19 unknown)	4	3.9%
Oligodendroglioma NEC (IDH1 wild type or 1p19q no codeleted)	6	5.8%
**Postsurgery treatment**		
Follow up	64	62.7%
RT	24	23.5%
RT + chemotherapy (PCV in all cases)	14	13.7%

^a^R132H mutation.

With respect to the type of surgery, 36 patients (35.3%) underwent complete resection, 34 patients (33.3%) were treated with partial resection, and only a biopsy procedure was performed in 32 patients (31.4%).

We classified the patients based on the WHO 2016 classification, and we added the concepts of the new WHO classification NOS (not otherwise specified, due to the diagnostic information, histological or molecular, necessary to assign a specific WHO diagnosis is not available, and NEC (not Elsewhere specified, which indicates that the necessary diagnostic testing has been successfully performed but that the results do not readily allow for a WHO diagnosis)^8^, 39 patients were astrocytoma IDH1 mutant, 14 astrocytoma IDH 1 wild type, 11 astrocytoma NOS, 28 patients were oligodendroglioma IDH1 mutant and 1p19q codeletion, 4 oligodendroglioma NOS and the remaining 6 patients were diagnosed oligodendroglioma NEC.

Following surgery, 62.7% of the patients did not undergo adjuvant treatment, 23.5% were treated with radiotherapy alone, and 13.7% received sequential chemotherapy/radiotherapy. Of those who received chemotherapy, the protocol was procarbacine-CCNU-vincristine (PCV) in all cases.

IDH1 mutational status was assessed by immunohistochemistry in 90 of 102 patients (88.2%), and the 1p19q codeletion status was determined in all patients with a histological diagnosis of oligodendroglioma.

After applying the different risk scores, according to the EORTC criteria, 56.9% (58/102) and 43.1% (44/102) of the patients were classified as low and high risk, respectively. By applying the RTOG criteria, only 28.4% (29/102) of the patients were considered to be in the low-risk group, and the remaining 71.6% (73/102) were assigned to the high-risk group.

### Degree of consistency between RTOG and EORTC criteria and risk classification according to clinical scores

The degree of consistency between the EORTC and RTOG criteria was low, 71.6% (K = 0.431; p = 0.0001).

Among those classified as high risk according to the EORTC criteria (n = 44), 97.7% were also considered to be at high risk by the RTOG criteria. In contrast, among those assigned to the low-risk group according to the EORTC score (n = 58), 51.7% were classified as high risk when applying the RTOG criteria.

Among the low-risk RTOG group (n = 29), only 45.3% were also deemed to be low risk according to EORTC, and among those high-risk patients according to RTOG score (n = 73), 51.7% were classified as low risk with EORTC criteria (Table 3).

**Table 3:** Degree of consistency between EORTC and RTOG criteria.

	RTOG		Total (n, %)
	Low risk (n, %)	High risk (n, %)	
**EORTC**			
Low risk	28/58 45.3%	30/58 51.7%	58/102 56.8%
High risk	1/44 2.3%	43/44 97.7%	44/102 43.1%
Total (n, %)	29/102 28.4%	73/102 71.6%	

### Survival analysis

With regard to the survival analysis in the different prognostic groups, in those assessed with the RTOG criteria, the median OS was 318 months for those classified as low risk and 91 months for those classified as high risk (p < 0.001).

After applying the EORTC criteria, the median OS was 223 months for those classified as low risk and 29 months for those classified as high risk (p < 0.00001).

In addition, overall survival was also assessed according to the type of surgery and IDH mutation status. In patients treated with complete surgery, OS was 318 months, compared to 191 months in those who underwent partial surgery and only 19 months for those who underwent only one biopsy procedure; these differences were highly significant (p = 0.0001).

The IDH mutational analyses revealed that 78% of patients harbored an IDH1 mutation (70/90). The OS among those bearing an IDH1 mutation was 192 months compared with 22 months in IDHwt patients (p = 0.0001).

When analyzing OS according to the 2016 WHO classification by adding the NOS (Not Otherwise Specified) and NEC (Not elsewhere Specified) subtypes of the new WHO classification without taking into account other factors, OS was higher in patients with oligodendroglioma, IDH mutation and 1p19q codeletion (n = 28; median not reached with a median follow-up of 191 months), followed by those classified as oligodendroglioma NEC (n = 6; OS 353 months), those with IDH mutant astrocytoma (n = 39; OS 174 months), and those with IDHwt astrocytoma (n = 14; OS 116 months). OS was lower in patients with oligodendroglioma NOS (n = 4; OS 114 months) and astrocytoma NOS (n = 9; OS 90 months), although the number of patients in these two groups is too small to draw conclusions. We also found that the functional status of patients measured by the ECOG scale was related to survival, and ECOG > 1 had a hazard ratio (HR) of 6.9 (95% CI 3.4–13.7, p < 0.001).

A univariate and multivariate analysis was performed using Cox regression in which age ≥ 40 years, size > 6 cm, surgical intervention with complete resection and the presence of IDH mutation were independent significant related factors. Afterwards, we included astrocytoma histology type because in other papers, it was related to mortality. In our model, astrocytoma histology type was not related to mortality in the multivariate or univariate analysis. We show these findings in Table 4.

**Table 4:** Prognostic factor univariant and multivariant analysis.

	Univariant			Multivariant		
	HR	95% CI HR	p	HR	95% CI HR	p
Age ≥ 40 years	3.72	1.99–6.97	< 0.001	3.57	1.77–7.19	< 0.001
Size > 6 cm	2.27	1.23–4.20	0.009	2.22	1.14–4.35	0.020
Complete resection	0.21	0.09–0.50	< 0.001	0.384	0.16–0.94	0.035
IDH mutation	0.29	0.16–0.51	< 0.001	0.373	0.19–0.71	0.003
Astrocytoma	1.63	0.89–3.01	0.114	1.41	0.70–2.85	0.335

We tried to build a parsimonious model (multivariate analysis) with the different variables (age, IDH mutation status, resection type, size > 6 cm, 1p19q codeletion status, histology, and RTOG and EORTC score). In this model, only IDH mutation and EORTC score were the factors that impacted prognostic prediction (Table 5).

**Table 5:** Analysis multivariant (parsimonious model).

	Univariant			Multivariant		
	HR	95% CI HR	p	HR	95% CI HR	p
EORTC	10.9	5.18–22.7	< 0.001	9.11	4.26–19.5	< 0.001
IDH mutation	0.29	0.16–0.51	< 0.001	0.46	0.25–0.84	0.011

According to our model, these two factors (IDH status and EORTC model) could be used together to elaborate a prognostic model, which implies a change in the indication for treatment instead of the currently most used RTOG score, which in our model is a factor that has no independent prognostic impact.

**Figure 1:**
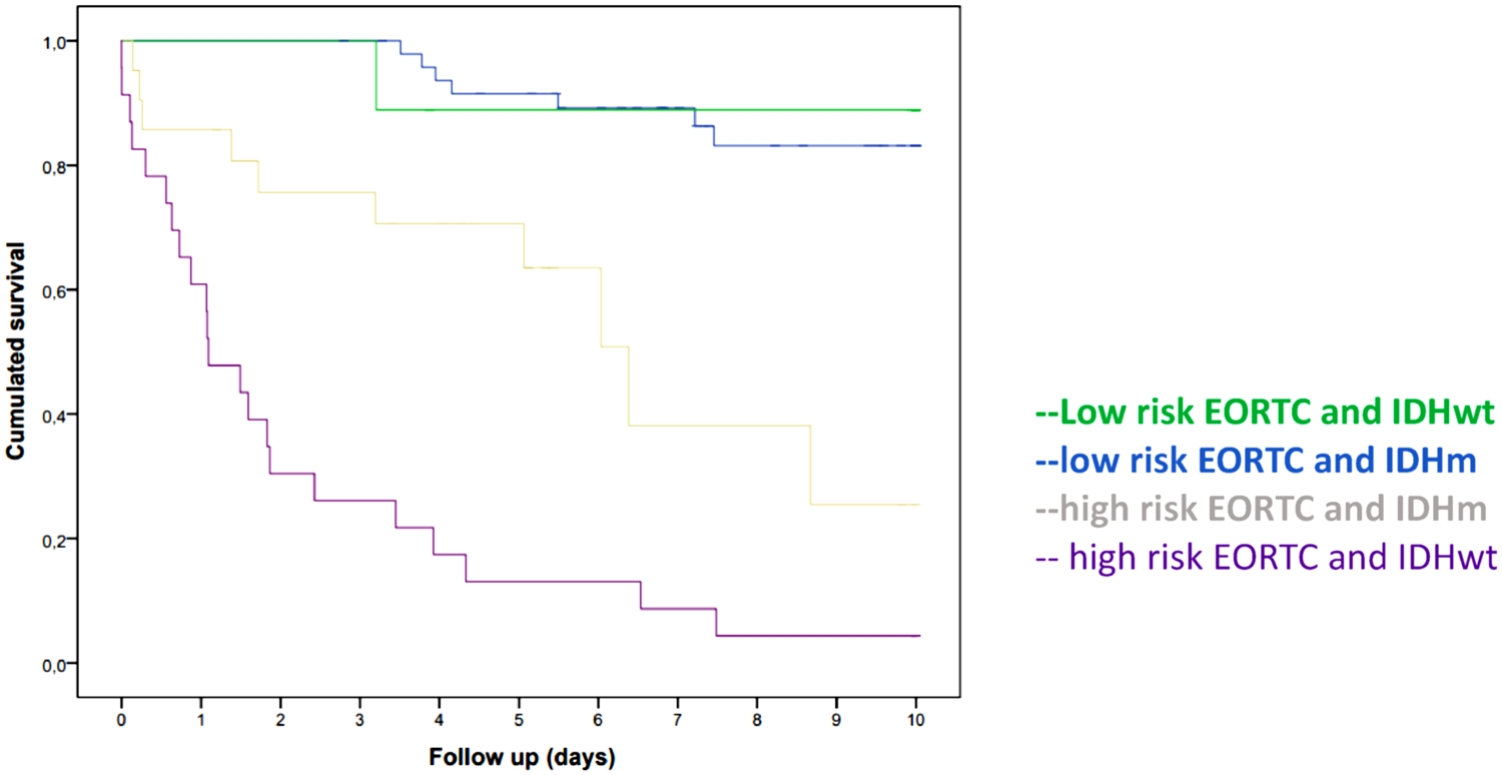
Overall survival analysis as a function of EORTC score and HDI status.

In addition, we present the survival analysis according to the EORTC score and HDI status. It is worth noting that in the low-risk EORTC group with IDH wt (Fig. 1).

## Discussion

Despite acknowledging the existence of great heterogeneity among LGGs, with a wide prognostic range, clinical and histological classification still remain the structural basis on which the diagnosis and subsequent treatment strategy are established in patients with LGG. Despite the great advances in the molecular understanding of glioma biology, which drastically changed the WHO histopathological classification of these tumors, clinical classifications, such as EORTC and RTOG scores, are still applied to decide which patients should undergo postoperative treatment with chemotherapy and/or radiotherapy. Indeed, adjuvant treatment algorithms based on these scores have been established^9,10^ and still serve as a guideline to indicate treatment. Currently, despite the lack of evidence of randomized studies evaluating adjuvant treatment based on molecular risk criteria, some guidelines are beginning to include them^11^, and our study clearly suggests the importance of these factors. Indeed, adjuvant treatment algorithms based on those scores have been established^9^, but no molecular markers are still included, even though the strong influence of some of these markers on prognosis, so they should be updated.

In 2016, the World Health Organization (WHO) updated the classification of CNS tumors, incorporating molecular biomarkers as part of the diagnosis of low-grade and high-grade gliomas, including mutations in the *isocitrate dehydrogenase 1 and 2* genes (IDH) and the 1p/19q codeletion^3^.

In the 2021 WHO 5th edition, it is contemplated to add the suffix NOS, which indicates that the information (histologic or molecular) to assign a specific WHO diagnosis is not available, and the NEC suffix, which indicates that diagnostic tests have been performed but the results do not allow a WHO diagnosis to be concluded; for example, a discordant result between histology, immunohistochemistry and/or genetics.

The better prognosis of IDH mutation carriers is well known, regardless of treatment^12,13^. Furthermore, 90% of IDH-mutated gliomas harbor a point mutation in IDH1 R132H, which can be identified with immunohistochemistry methods, a simple and reproducible method that can be performed in the vast majority of pathology departments, but for the 10% of patients with IDH2 mutation, molecular techniques are not available in most hospitals^14^.

The classical EORTC prognostic factors were described by Pignatti in 2002^7^ after performing a retrospective analysis of two phase III trials (EORTC 22844–22845) evaluating the role of radiotherapy in LGGs. Multivariate analysis showed that age ≥ 40 years, astrocytoma histology, maximum tumor diameter ≥ 6 cm, tumors crossing the midline, and the presence of neurological deficits prior to surgery were adverse factors for survival. Thus, a prognostic model was established in which the overall survival (OS) curves were separated according to the number of unfavorable factors present in each patient. After classifying patients into two risk categories (low risk: 0–2 factors and high risk: 3–5 factors), the median OS in the validation group was 7.8 and 3.7 years, respectively. This is the most widely accepted clinical classification because it is based on patients included in EORTC clinical trials, who were prospectively included on the basis of uniform inclusion criteria.

RTOG only takes into account two prognostic factors: age ≥ 40 and absence of complete resection after surgery. These criteria were adopted as inclusion criteria in the RTOG 9802 trial, a trial that demonstrated an increase in OS and PFS with sequential addition of chemotherapy to radiotherapy (median OS 13.3 years for treatment with sequential radiation and chemotherapy versus 7.8 years in patients receiving radiotherapy alone)^3^. Based on this study, all patients diagnosed with LGG with any of these two adverse prognostic factors (incomplete surgery or ≥ 40 years) should be offered adjuvant treatment.

However, the molecular analysis of IDH was not performed prospectively in EORTC or RTOG studies, and there is controversy about the implication of each factor in the prognosis of LGG. In fact, Gorlia et al.^15^ in 2013 performed an analysis of 339 patients who had participated in the 2 EORTC trials (22844/22845), including only grade II tumors, and observed that pre-surgery neurological deficit, symptom interval, astrocytic lineage and size > 5 cm were adverse prognostic factors for progression-free survival (PFS) and for overall survival (OS), whereas early treatment with radiotherapy was only prognostic in terms of PFS. In this study, neither age nor extent of surgery had a significant prognostic impact. However, age is also a recognized prognostic factor in other scoring scales, and Lote et al. reported better survival at younger ages in 1997^12^.

Currently, according to the current WHO classification, molecular profiling that includes evaluation of IDH mutational status and 1p19q codeletion should be performed in LGG assessment, although it is unknown how to integrate these into the scores to help clinicians decide whether or not adjuvant treatment should be offered, with the RTOG criteria prevailing in the algorithm.

In the present study, the multivariate analysis clearly established the type of resection and the presence of IDH mutation as independent prognostic factors for OS. As in the study by Gorlia et al.^15^, age was not an independent prognostic factor either, which contrasts with EORTC criteria and former scoring scales reporting better survival in younger ages^16^. Furthermore, when EORTC and RTOG criteria were included in the multivariate analysis, the RTOG criteria did not independently correlate with OS, but the EORTC criteria did.

In the present study, the degree of consistency between the EORTC and RTOG criteria was 71.6% (K = 0.426; p = 0.0001), higher than that described by Franceschi et al.^17^ in a similar analysis. In addition, among patients classified as high risk by EORTC criteria (n = 44), 97.7% were also considered to be high-risk LGG after RTOG criteria, and the same was true for those labeled as low risk by RTOG criteria (n = 29); 96.6% were also classified as low risk according to EORTC criteria. However, a strong discrepancy was observed among those classified as low risk by EORTC criteria (n = 58) because in this group, almost 52% (30/58) of patients were classified as high risk according to the RTOG criteria. This is because the RTOG considers any type of incomplete resection to be high risk, whereas the EORTC does not include the type of resection in the score.

Additionally, in the present study, both classifications (RTOG and EORTC) were able to differentiate prognostic groups with a significant impact on OS between those classified as low or high risk, even though the gap between low- and high-grade curves was larger with the EORTC criteria, which could result in a better selection of low-grade patients. Nonetheless, it could be pointed out that, although the EORTC does not include complete resection among its factors, midline involvement and, to a lesser extent, tumor size ≥ 6 cm preclude the achievement of complete resection.

The presence of IDH mutations and 1p19q codeletion has also been shown to be an independent prognostic factor for OS; therefore, it should be introduced in the risk scores to decide which patients should receive complementary treatment after surgery.

Prior to 2016, the classification of CNS tumors was based on histological features, and we know that certain molecular markers can provide powerful prognostic information. Therefore, in the recent WHO classification proposal, molecular parameters have been added for grading and prognostic estimation, such as homozygous deletion of CDKN2A/B in IDH-mutated astrocytomas, TERT promoter mutation, EGFR amplification and + 7/−10 copy number changes in IDH wild-type diffuse astrocytomas (allowing a glioblastoma designation, even in cases that otherwise appear histologically to be lower grade).

The main limitations of our study are its retrospective nature and the limited determination of molecular alterations because we only performed IHC analysis of IDH 1 and 1p19q in patients categorized as OLigodendroglioma.

IDH mutations (in our center, only IDH1 is determined by IHC of IDH1-R132H), which reflects the profile of the majority of healthcare centers in our country.

The problem is the implementation of these techniques in the care setting, as they are not available in many hospitals, so we must be clear about which of these markers will guide us in the treatment, and our proposal would be clinical staging (EORTC) and the determination of IDH and 1p19q as a minimum recommendation.

## Conclusion

The degree of consistency between the RTOG and EORTC risk scores in this study population was low (71.6%). Although the two classifications were able to differentiate risk groups with a significant impact on OS, a larger discrepancy was observed among patients labeled as low risk by EORTC, as more than 50% of these were classified as high risk by RTOG, with consequent adjuvant therapeutic implications. Moreover, in a multivariate analysis, the presence of IDH mutation, the type of surgery and the EORTC criteria were all independent prognostic factors for OS, whereas the RTOG criteria were not.

## References

[1] Morshed RA, Young JS, Hervey-Jumper SL, Berger MS (2019). The management of low-grade gliomas in adults. J. Neurosurg. Sci..

[2] Pouratian N, Asthagiri A, Jagannathan J, Shaffrey ME, Schiff D (2007). Surgery Insight: The role of surgery in the management of low-grade gliomas. Nat. Clin. Pract. Neurol..

[3] Louis DN, Perry A, Reifenberger G, von Deimling A, Figarella-Branger D, Cavenee WK (2016). The 2016 World Health Organization classification of tumors of the central nervous system: A summary. Acta Neuropathol. (Berl)..

[4] Buckner JC, Shaw EG, Pugh SL, Chakravarti A, Gilbert MR, Barger GR (2016). Radiation plus Procarbazine, CCNU, and Vincristine in low-grade glioma. N. Engl. J. Med..

[5] van den Bent MJ, Afra D, de Witte O, Ben Hassel M, Schraub S, Hoang-Xuan K (2005). Long-term efficacy of early versus delayed radiotherapy for low-grade astrocytoma and oligodendroglioma in adults: the EORTC 22845 randomised trial. Lancet Lond. Engl..

[6] Cancer Genome Atlas Research Network, Brat, D.J., Verhaak, R.G.W., Aldape, K.D., Yung, W.K.A., Salama, S.R., *et al*. Comprehensive, integrative genomic analysis of diffuse lower-grade gliomas. *N. Engl. J. Med*. **372**(26), 2481–2498 (2015). 10.1056/NEJMoa1402121 PMC4530011 26061751

[7] Eckel-Passow JE, Lachance DH, Molinaro AM, Walsh KM, Decker PA, Sicotte H (2015). Glioma groups based on 1p/19q, IDH, and TERT promoter mutations in tumors. N. Engl. J. Med..

[8] Louis DN, Perry A, Wesseling P, Brat DJ, Cree IA, Figarella-Branger D (2021). The 2021 WHO Classification of Tumors of the central nervous system: A summary. Neuro-Oncol..

[9] Soffietti, R., Baumert, B.G., Bello, L., Grisold, W., Grant, R., Graus, F., *et al*. *Guidelines on the Management of Low-Grade Gliomas: EANO Task Force Report*. Vol. 9. 10.1111/j.1468-1331.2010.03151.x 20718851

[10] Ryckman JM, Appiah AK, Lyden E, Verma V, Zhang C (2019). Concurrent versus sequential chemoradiation for low-grade gliomas meeting RTOG 9802 criteria. Am. J. Clin. Oncol..

[11] cns.pdf [Internet]. Disponible en https://www.nccn.org/professionals/physician_gls/pdf/cns.pdf. Accessed 21 Jan 2022 (2022).

[12] Houillier C, Wang X, Kaloshi G, Mokhtari K, Guillevin R, Laffaire J (2010). IDH1 or IDH2 mutations predict longer survival and response to temozolomide in low-grade gliomas. Neurology.

[13] Yan H, Parsons DW, Jin G, McLendon R, Rasheed BA, Yuan W (2009). IDH1 and IDH2 mutations in gliomas. N. Engl. J. Med..

[14] Poetsch L, Bronnimann C, Loiseau H, Frénel JS, Siegfried A, Seizeur R (2021). Characteristics of IDH-mutant gliomas with non-canonical IDH mutation. J. Neurooncol..

[15] Gorlia T, Wu W, Wang M, Baumert BG, Mehta M, Buckner JC (2013). New validated prognostic models and prognostic calculators in patients with low-grade gliomas diagnosed by central pathology review: A pooled analysis of EORTC/RTOG/NCCTG phase III clinical trials. Neuro-Oncol..

[16] Lote K, Egeland T, Hager B, Stenwig B, Skullerud K, Berg-Johnsen J (1997). Survival, prognostic factors, and therapeutic efficacy in low-grade glioma: A retrospective study in 379 patients. J. Clin. Oncol. Off. J. Am. Soc. Clin. Oncol..

[17] Franceschi E, Mura A, Lamberti G, De Biase D, Tosoni A, Di Battista M (2019). Concordance between RTOG and EORTC prognostic criteria in low-grade gliomas. Future Oncol. Lond. Engl..

